# Reactivation from latency displays HIV particle budding at plasma membrane, accompanying CD44 upregulation and recruitment

**DOI:** 10.1186/1742-4690-6-63

**Published:** 2009-07-13

**Authors:** Mari Suyama, Eriko Daikoku, Toshiyuki Goto, Kouichi Sano, Yuko Morikawa

**Affiliations:** 1 Kitasato University, Shirokane 5-9-1, Minato-ku, Tokyo 108-8641, Japan; 2 Osaka Medical College, Daigaku-cho 2-7, Takatsuki, Osaka 569-8686, Japan; 3 School of Health Science, Faculty of Medicine, Kyoto University, Kawaraha-cho 53, Shogoin, Sakyo-ku, Kyoto 606-8507, Japan

## Abstract

**Background:**

It has been accepted that HIV buds from the cell surface in T lymphocytes, whereas in macrophages it buds into intracellular endosomes. Recent studies, on the other hand, suggest that HIV preferentially buds from the cell surface even in monocytic cells. However, most studies are based on observations in acutely infected cells and little is known about HIV budding concomitant with reactivation from latency. Such studies would provide a better understanding of a reservoir for HIV.

**Results:**

We observed HIV budding in latently infected T lymphocytic and monocytic cell lines following TNF-α stimulation and examined the upregulation of host factors that may be involved in particle production. Electron microscopy analysis revealed that reactivation of latently infected J1.1 cells (latently infected Jurkat cells with HIV-1) and U1 cells (latently infected U937 cells with HIV-1) displayed HIV particle budding predominantly at the plasma membrane, a morphology that is similar to particle budding in acutely infected Jurkat and U937 cells. When mRNA expression levels were quantified by qRT-PCR, we found that particle production from reactivated J1.1 and U1 cells was accompanied by CD44 upregulation. This upregulation was similarly observed when Jurkat and U937 cells were acutely infected with HIV-1 but not when just stimulated with TNF-α, suggesting that CD44 upregulation was linked with HIV production but not with cell stimulation. The molecules in endocytic pathways such as CD63 and HRS were also upregulated when U1 cells were reactivated and U937 cells were acutely infected with HIV-1. Confocal microscopy revealed that these upregulated host molecules were recruited to and accumulated at the sites where mature particles were formed at the plasma membrane.

**Conclusion:**

Our study indicates that HIV particles are budded at the plasma membrane upon reactivation from latency, a morphology that is similar to particle budding in acute infection. Our data also suggest that HIV expression may lead to the upregulation of certain host cell molecules that are recruited to sites of particle assembly, possibly coordinating particle production.

## Findings

It has been thought that HIV particles assemble and bud at the plasma membrane (PM) in T lymphocytes and HeLa cells, but at the endosomes in macrophages, suggesting that such endosomal targeting may be essential for HIV budding in macrophages [1-6]. However, recent studies using the inhibitors of the endocytic pathway and membrane-impermeant dyes have revealed that the PM is the primary site for HIV assembly and particle budding even in macrophages and that particles accumulate at the endosomes through endocytosis [7-9]. Nevertheless, these studies are based on observations in acutely infected cells and little is known about HIV budding concomitant with reactivation from latency. Latently infected resting T cells are known to serve as a stable reservoir for HIV during anti-retroviral therapy and to produce infectious particles upon cell reactivation. Studies on HIV production from latently infected cells upon reactivation are necessary for a better understanding of HIV pathogenesis, although some studies have indicated intracellular accumulation of particles in chronically or latently infected cells [10,11]. Here, we employed J1.1 cells that were Jurkat T lymphocytic cells latently infected with HIV-1, and U1 cells that were U937 monocytic cells latently infected with HIV-1, and observed HIV particle budding following reactivation.

**Figure 1:**
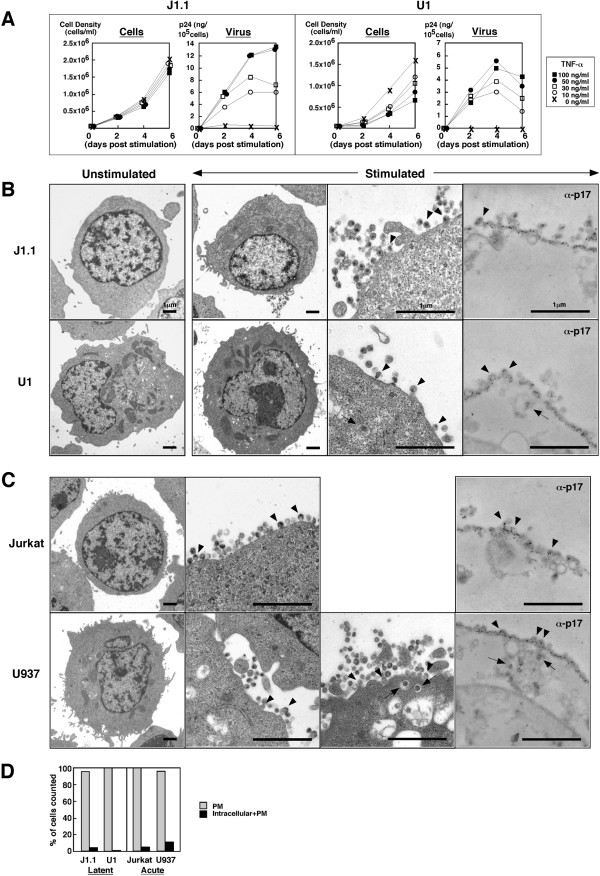
**Reactivation of latently infected J1.1 and U1 cells displays HIV particle budding at the PM**. (A) HIV production from J1.1 and U1 cells upon TNF-α stimulation. J1.1 and U1 cells were stimulated with TNF-α (~100 ng/ml). Levels of particle production were measured by p24 antigen ELISA. (B) HIV particle budding from J1.1 and U1 cells upon TNF-α stimulation. J1.1 and U1 cells stimulated with 50 ng/ml TNF-α were subjected to conventional electron microscopy and immunoelectric microscopy using anti-HIV-1 p17MA antibody. (C) HIV particle budding from acutely infected Jurkat and U937 cells. Jurkat and U937 cells were infected with HIV-1 (LAV strain) corresponding to 100–200 ng of p24CA antigen and were analyzed by electron microscopy. Arrowheads indicate budding particles and arrows indicate particles into intracellular vesicles in (B) and (C). (D) Semi-quantification of HIV-1 particle localization. Approximately 300 of particle-positive cells observed by conventional electron microscopy were sorted into the categories indicated.

We initially tested the dose of TNF-α, and temporally monitored cell growth and HIV particle production after stimulation (Fig. 1A). J1.1 cells proliferated equally regardless of the dose of TNF-α, and the particle production levels increased to 50 ng/ml TNF-α. In contrast, proliferation of U1 cells was inhibited in a dose-dependent manner, and the highest level of particle production was observed at 50 ng/ml. We thus used 50 ng/ml TNF-α for further experiments. To avoid nonspecific stimulation by changing the medium, we added TNF-α directly to the culture medium, and this led to the higher dose of TNF-α required in our study than in other reports [12,13].

Electron microscopy was carried out to examine where particle budding occurred in J1.1 and U1 cells upon reactivation (Fig. 1B). Little or no particles were produced in either cell line before TNF-α stimulation (Fig. 1B, most left panels), consistent with previous reports [11-14]. Upon stimulation, nascent budding particles were visible on the surface of nearly all J1.1 cells, similar to the case with U1 cells (Fig. 1B, arrowheads). Unexpectedly, particles in intracellular vesicles were rarely seen in both J1.1 and U1 cells (Fig. 1B, arrow). The findings were confirmed by immunoelectric microscopy using anti-HIV-1 p17MA antibody (Fig. 1B, most right panels). Next, their parental cell lines, Jurkat and U937 cells, were infected with HIV-1, and particle production in acute infection were examined by electron microscopy. Particle budding was observed predominantly at the PM of both Jurkat and U937 cells (Fig. 1C, arrowheads) but some U937 cells displayed budding into intracellular compartments (Fig. 1C, arrows). Immunoelectric microscopy indicated similar results (Fig. 1C, most right panels). For quantification, we counted the number of cells containing particles at the PM alone or that of cells containing particles at both intracellular vesicles and the PM (Fig. 1D). Budding at the PM was prominent, regardless of whether cells were acutely or latently infected, or T lymphocytic or monocytic, suggesting that unlike chronically infected cells [10], HIV particles are most likely budded from the PM in latently infected cells, although it cannot be ruled out in this experiment that the particles observed in extracellular spaces might be released by exocytosis.

**Figure 2:**
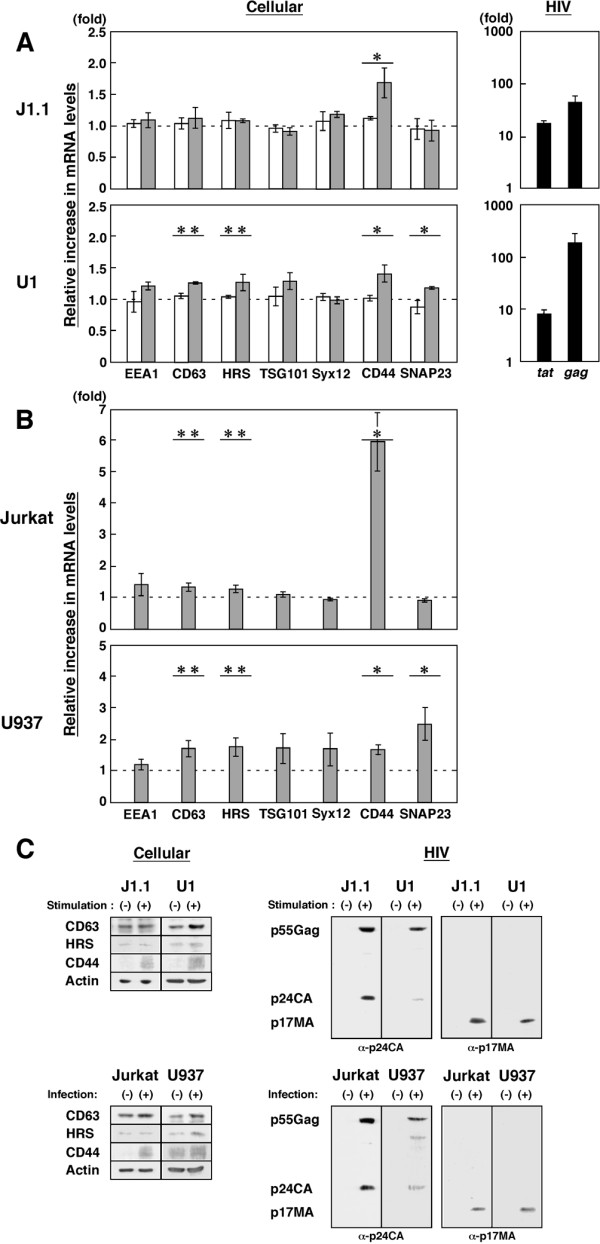
**HIV particle production is accompanied by mRNA upregulation of CD44 and endocytic molecules**. (A) Differential gene expression upon reactivation. J1.1 and U1 cells were either unstimulated or stimulated with 50 ng/ml TNF-α (gray columns). For comparison, uninfected Jurkat and U937 cells were similarly stimulated (white columns). Expression of each gene was quantified by qRT-PCR and normalized to the level of GAPDH. The fold increase of expression of each gene upon stimulation was shown. *, p < 0.01; **, p < 0.05. Expression levels of HIV-1 *gag *and *tat *mRNAs were quantified using specific primers (HIV-1 nucleotide positions 701–720 and 787–806 for *gag *and 5965–5987 and 8389–8411 for *tat*, respectively) (black columns). (B) Differential gene expression upon acute infection. Jurkat and U937 cells were infected with HIV-1 and subjected to qRT-PCR. The fold increase of each gene expression upon infection was shown (gray columns). *, p < 0.01; **, p < 0.05. (C) Protein expression of J1.1, U1, Jurkat, and U937 cells. TNF-α stimulation and infection were similarly performed. Cells were subjected to Western blotting using anti-CD44, anti-CD63, anti-HRS, anti-actin, anti-HIV-1 p17MA, and anti-p24CA antibodies.

Gene expression analysis based on cDNA microarrays has extensively been employed and has provided evidence for the modulation of host cellular gene expression upon HIV infection (replication and latency) [15-20]. Although numerous host genes are modulated upon HIV infection, it is conceivable that expression levels of host membrane components may change by feedback regulation upon HIV reactivation, as HIV requires host cell membrane for particle budding. A membrane contains a number of microdomains, enriched in cholesterol (i.e., rafts) and in tetraspanins (e.g., CD63 and CD81), which accumulate at sites of HIV budding [7,21-26]. It has been shown that TSG101, a component of endosomal sorting complex required for transport (ESCRT) is recruited to the sites of particle assembly and is responsible for HIV particle budding [27,28]. Thus we chose endosomal (EEA1, CD63, HRS, TSG101, and Syntaxin12) and PM (CD44 and SNAP23) markers and quantified their mRNA levels by qRT-PCR (Fig. 2A and 2B) using the primer sets shown in Additional File 1. Their properties and functions are as follows: EEA1 is a marker molecule for early endosome; HRS is an initial molecule for the ESCRT pathway; Syntaxin12 is a SNARE molecule for endosomal membrane fusion; CD44 is an adhesion molecule implicated in cell migration; SNAP23 is a SNARE molecule for PM fusion in the exocytic pathway. When the mRNA levels in J1.1 cells stimulated with TNF-α were compared with those in unstimulated J1.1 cells, CD44 gene expression was increased, but the other genes tested were largely unaltered. No significant upregulation of CD44 was observed when cells of its uninfected parental line, Jurkat, were similarly stimulated with TNF-α, indicating that the CD44 upregulation was not simply due to cell stimulation (Fig. 2A, upper). CD44 has been reported hardly expressed even at mRNA level in unstimulated Jurkat cells [29]. A similar analysis was carried out for U1 cells. Downregulation of CD44 has been reported for chronically infected monocytic cells [30]. Upon reactivation, CD44 upregulation was apparent but the endocytic molecules (CD63 and HRS) and SNAP23 were also upregulated in U1 cells. The modulation of others such as TSG101 was not statistically significant. These upregulations were not observed when uninfected U937 cells were stimulated (Fig. 2A, lower). The gene expression profiles upon reactivation were consistent with protein expression levels of the molecules when analyzed by Western blotting (Fig. 2C). We cannot simply compare the data of J1.1 and U1 cells, since expression levels of individual genes differ between the cell lines, but these significant upregulations were not observed in their parental but uninfected cells, suggesting that the upregulations might be linked with HIV expression. To test this possibility, we quantified expression levels of the same genes in acutely infected Jurkat and U937 cells and compared them with the levels in uninfected Jurkat and U937 cells. Upregulation of CD44 was observed in acutely infected Jurkat cells (Fig. 2B, upper), and this magnitude fold of upregulation was likely due to a very low level of CD44 expression in uninfected Jurkat cells [29]. In acutely infected U937 cells, besides CD44 upregulation, upregulation of other genes (CD63, HRS, and SNAP23) was observed (Fig. 2B, lower). Together, the results indicate that the upregulation of host molecules observed here was likely to be linked with HIV production. Higher levels of *gag *mRNA than *tat *mRNA observed in this study were possibly because we analyzed at a late stage of HIV replication. Western blotting confirmed HIV antigens, p55Gag precursor and its processing products, p24CA and p17MA, appeared upon reactivation or infection and showed that unlike anti-p24CA antibody, anti-p17MA antibody used in this study (against the C-terminal region of p17MA) recognized the mature p17MA domain but not the unprocessed p55Gag (Fig. 2C).

**Figure 3:**
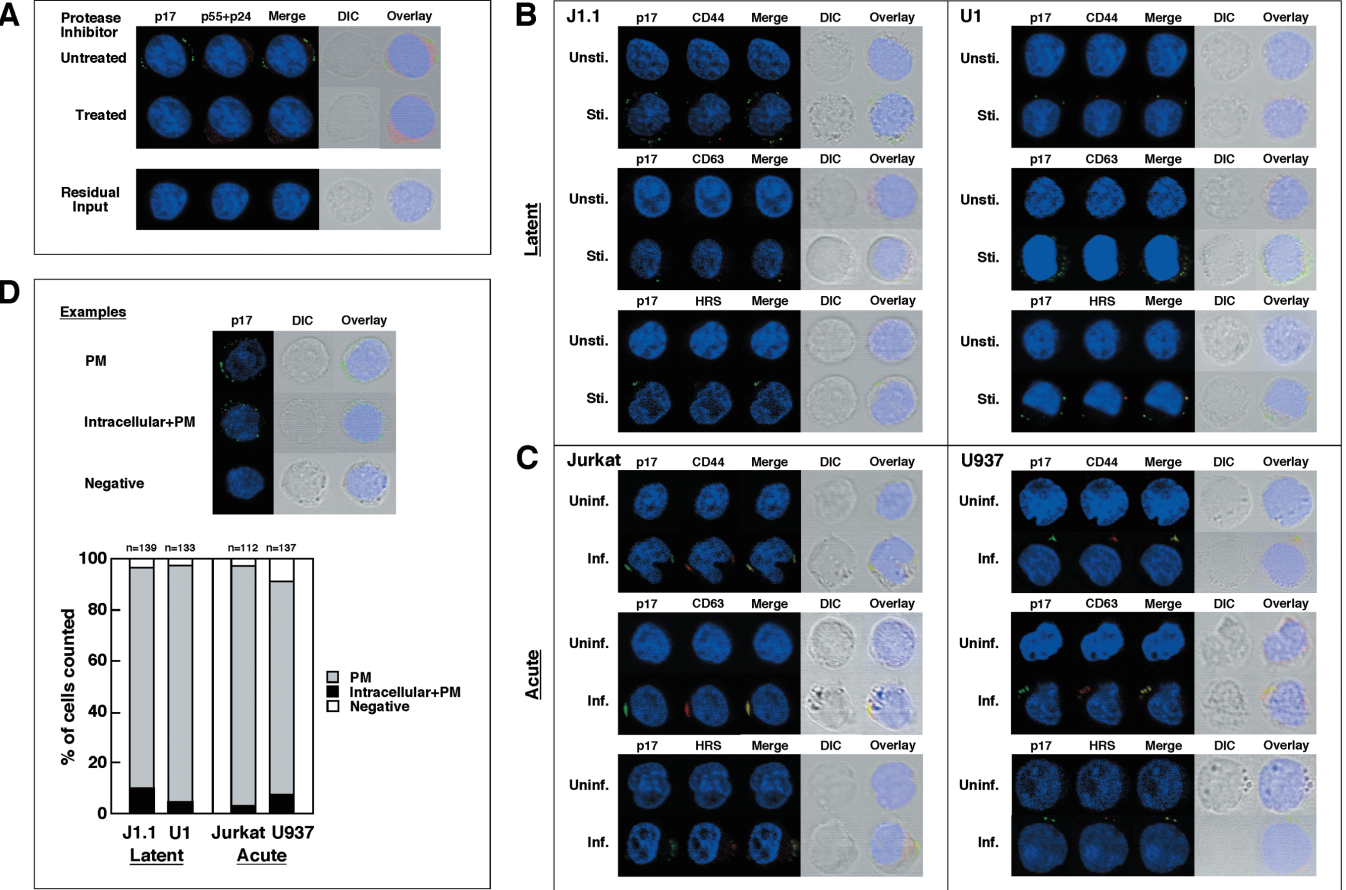
**Upregulated molecules are accumulated to sites of HIV-1 particle budding**. (A) Inhibition of Gag processing in J1.1 cells stimulated but treated with 1 μM ritonavir (upper) and residual HIV in Jurkat cells after infection (lower). For confocal microscopy, the cells were stained with anti-HIV-1 p17MA (green), p24CA (red) antibodies and with TOPRO-3 (blue). (B) Intracellular localization of upregulated molecules upon reactivation. J1.1 and U1 cells were either unstimulated (Unsti.) or stimulated (Sti.) with TNF-α and were immunostained with anti-p17MA antibody (green) and antibodies for CD44, CD63, and HRS (red). (C) Intracellular localization of upregulated molecules upon acute infection. Jurkat and U937 cells were infected with HIV-1 and immunostained. Inf., infected; Uninf., uninfected. (D) Semi-quantification of sites for HIV-1 particle production. Examples of cells exhibiting PM staining alone, intracellular+PM accumulations, and no signals (negative) (upper). Based on p17MA localization (PM, intracellular+PM, or negative), approximately 100–150 cells were sorted into the categories (lower).

Confocal microscopy revealed that the anti-p17MA antibody specifically detected mature p17MA produced upon HIV protease-mediated Gag processing (Fig. 3A). Since Gag processing occurs concomitant with particle budding, the p17MA signal obtained with the antibody most likely represents the sites of particle budding [4,31]. No p17MA signal was seen on the day after infection, indicating that it was not derived from residual HIV (Fig. 3A). When J1.1 and U1 cells were reactivated, the p17MA antigens were observed at the cell periphery, likely at the PM, but no signals were seen in unstimulated cells (Fig. 3B). Similarly, the p17MA antigens were observed at the PM in acutely infected Jurkat and U937 cells (Fig. 3C). For quantification, we counted the numbers of cells based on p17MA distribution patterns (PM, intracellular+PM, or negative) and confirmed that HIV particles were preferentially formed at the PM (Fig. 3D), consistent with the data obtained by electron microscopy (Fig. 1D).

To understand the significance of the upregulation of host molecules observed here, we examined intracellular localization of the molecules by immunostaining. No CD44 staining was found in unstimulated J1.1 and U1 cells, consistent with previous reports indicating CD44 downregulation during latency [30,32]. Following reactivation, CD44 was visible and colocalized with the p17MA antigens at the PM. Similarly, CD63 and HRS stainings were rarely seen in unstimulated cells but became visible and colocalized with the 17MA signals, especially in U1 cells (Fig. 3B). These findings were very apparent in acutely infected cells (Fig. 3C): CD63 recruitment, as reported previously for acutely infected Jurkat T cells and macrophages [7,25,33,34], and HRS and CD44 accumulations to the sites where mature particles were formed. Together, our data suggest that HIV expression may lead to the upregulation of certain host molecules that are recruited to the sites of particle assembly, possibly to coordinate particle production. Because CD44 is a cell adhesion molecule that mediates lymphocyte aggregation and homing [35,36], it is conceivable that the CD44 recruitment to HIV assembly sites may lead to an efficient cell-to-cell transmission of HIV and infected cell migration to lymph nodes.

In conclusion, despite numerous literature on HIV budding to intracellular compartments especially in macrophages, our data indicate that upon reactivation from latent infection, HIV predominantly buds at the PM, a morphology that is similar to particle budding in acute infection, suggesting that HIV latency have a potential for robust production of HIV observed for acute infection.

## Abbreviations

HIV: human immunodeficiency virus; TNF-α: tumor necrosis factor-α; CA: capsid; MA: matrix; qRT-PCR: quantitative RT-PCR; TSG101: tumor susceptibility gene-101; HRS: hepatocyte growth factor regulated tyrosine kinase substrate; EEA1: early endosomal antigen 1; SNAP23: synaptosome associated 23 kDa protein.

## Competing interests

The authors declare that they have no competing interests.

## Authors' contributions

MS performed the qRT-PCR analysis and confocal study. ED, TG, and KS carried out the electron microscopy analysis. YM designed the experiment and wrote the manuscript.

## Supplementary Material

Additional file 1 **Sequences of primer sets used in the study.** Click here for file
