# Supplementary material for: Reactivation from latency displays HIV particle budding at plasma membrane, accompanying CD44 upregulation and recruitment
Source: Retrovirology. 2009 Jul 13;6:63. doi: 10.1186/1742-4690-6-63 (PMC2714482; doi:10.1186/1742-4690-6-63)
Supplement: Additional file 1 — Sequences of primer sets used in the study. [file 1742-4690-6-63-S1.doc]

Additional File 1

Sequences of primer sets used in the study

Gene Sequence Product Size (bp) Melting Temperature (°C)

HIV-1 gag Forward 5’-GCTTGCTGAAGCGCGCACGG-3’ 106 70.5

Reverse 5’-GACGCTCTCGCACCCATCTC-3’ 66.0

HIV-1 tat Forward 5’-TCCTATGGCAGGAAGAAGCGGAG-3’ 123 67.0

Reverse 5’-TTCCTTCGGGCCTGTCGGGTCCC-3’ 72.5

EEA1 Forward 5’-CCTGATGGGTTGGTGACTGATTC-3’ 136 65.0

Reverse 5’-CAGTAGCAAGTTGGGCTGCTTTC-3’ 64.5

CD63 Forward 5’-CTGGACAGGATGCAGGCAGA-3’ 121 64.9

Reverse 5’-CCACAGTAACATTAATGCAGCAGCAGGA-3’ 64.4

HRS Forward 5’-TCGCCTTGTATGCCCTGGA-3’ 82 65.0

Reverse 5’-TGCTTGTTGGCCACCTCATC-3’ 64.5

TSG101 Forward 5’-GTCCATATCCTGCCACAACAAGTTC-3’ 87 65.0

Reverse 5’-CGCTGATTGTGCCATCCCTAC-3’ 65.0

Syntaxin12 Forward 5’-TGACAGCCATGAGGAGTGGA-3’ 68 64.0

Reverse 5’-AATCCTGCTCAGTGATGGCC-3’ 63.0

CD44 Forward 5’-AACCCTTGCAACATTGCCTGA-3’ 80 65.0

Reverse 5’-GCTTCCAGAGTTACGCCCTTGA-3’ 65.0

SNAP23 Forward 5’-TTTCTGTTGAGGGCCGACTG-3’ 166 63.6

Reverse 5’-GGCTGTCTTTGGCAATGCTTC-3’ 64.3

GAPDH Forward 5’-GCACCGTCAAGGCTGAGAAC-3’ 138 63.3

Reverse 5’-TGGTGAAGACGCCAGTGGA-3’ 64.0
